# Transcriptomic and metabolomic profiling of *Polygonatum cyrtonema* Hua across different growth years

**DOI:** 10.3389/fpls.2026.1860542

**Published:** 2026-07-01

**Authors:** Jujia Zheng, Weijie Huang, Chun Chen, Pianpian Chen

**Affiliations:** Department of Pharmacy, Wenzhou Hospital of Integrated Traditional Chinese and Western Medicine affiliated to Zhejiang Chinese Medical University, Wenzhou, Zhejiang, China

**Keywords:** different growth years, differential metabolites, differentially expressed genes, gene, *Polygonatum cyrtonema* Hua, Metabolomic

## Abstract

*Polygonatum cyrtonema* Hua is a plant whose rhizome is famously used in traditional Chinese medicine. It has a wide range of pharmacological effects from the variety of active ingredients it contains, such as polysaccharides, flavonoid, and saponins. The increasing demand for *P. cyrtonema* is driven by growing public awareness of health. Artificial cultivation is the primary source of *P. cyrtonema*. Integrated transcriptomic and metabolomic analyses of biennial and triennial *P. cyrtonema* were used to investigate the impact of various growth years on the physiological mechanism of this plant. Using transcriptome analysis, 1721 differentially expressed genes (DEGs) were identified, comprising 1057 down-regulated and 664 up-regulated genes. The enrichment results of the Kyoto Encyclopedia of Genes and Genomes (KEGG) and other databases clarified the primary functions and enrichment sites of these DEGs, among which the phenylpropanoid biosynthetic pathway was the most crucial enrichment pathway. Additionally, 150 differential metabolites (DEMs) were derived from the metabolomics study, encompassing 78 down-regulated and 72 up-regulated metabolites. They underwent KEGG enrichment analysis in both POS and NEG modes. Through these different analyses, the common enrichment pathways-cofactors, glyoxylate and dicarboxylate metabolism, and carbohydrate metabolism were determined to be shared by them. Furthermore, several significant transcription factor families and pathways that are directly linked to the development of *P. cyrtonema* were discovered, including the bHLH, MYB, and NAC transcription factor families. Through in-depth study and analysis of these genes, their distinct roles in *P. cyrtonema* of various ages can be understood. This further suggests that growth years are one of the crucial factors affecting *P. cyrtonema* growth and development, active substance accumulation, and stress resistance.

## 1 Introduction

*P. cyrtonema* is a kind of traditional Chinese medicine. Numerous active ingredients, including polysaccharides, steroidal saponins, flavonoids, alkaloids, and others, provide *P. cyrtonema* a wide range of pharmacological actions (Lu et al., 2023). The Chinese Pharmacopoeia 2020 edition states that it can moisten the lung, tonify the kidney, replenish qi, and nourish Yin (Hu et al., 2022). Zhejiang, Anhui, Hubei, and other southern regions are where *P. cyrtonema* is primarily produced. These plants flourish in forests and other moist environments. This species is a Chinese medicinal herb possessing ornamental, therapeutic, and edible properties.

According to certain studies, one of the elements influencing the quality and active ingredient concentration of *P. cyrtonema* is the growth years (Zhao et al., 2018). In this study, transcriptomic and metabolomic analyses of biennial and triennial *P. cyrtonema* were analyzed to identify the differentially expressed genes (DEGs) and differential metabolites (DEMs). Analysis of the roles of DEGs and DEMs reveals their relationship with growth and development, allowing for better understanding of the mechanisms underlying the growth and development of *P. cyrtonema* at different ages.

The transcriptome, the sum of all the RNAs that a living cell can produce, is a key tool for understanding cell phenotype and function. Transcriptomics primarily examines the status and control of gene transcription in cells at the entire level and gene expression at the RNA level. The study of stress resistance, growth and development, breeding, and other botanical topics, makes extensive use of transcriptome sequencing technologies. It is a crucial technique to enable investigation into critical genes and pathways along with differential expression (Aliche et al., 2021; Huang et al., 2023).

By displaying the impact of intrinsic or extrinsic stimuli on metabolism through the quantitative measurement of small molecules (molecular weight < 1000 Da), metabonomics presents the sequence of biological processes that take place during a certain physiological process (Castro-Moretti et al., 2020). The rapid advancement of nuclear magnetic resonance (NMR), liquid chromatography-mass spectrometry (LC-MS), gas chromatography-mass spectrometry (GC-MS), and other detection methods have made metabonomics a potent tool for studying abiotic stress responses, diversity, growth, and developmental regulation in botany (Bowling and Thomas, 2014; Ribbenstedt et al., 2018; Barros et al., 2020; Deng et al., 2020; Wang et al., 2021).

## 2 Materials and methods

### 2.1 Materials

*P. cyrtonema* that are 2 and 3 years old were used in this study. Each group contained 3 plants and were obtained from Chizhou Anhui. Samples were collected from the leaves and rhizomes of each group. The leaf samples were used for transcriptome sequencing.

### 2.2 RNA sequencing

Total RNA was extracted from leaf tissues use Omega kit to extract RNA according to its instructions. The concentration and integrity of the extracted RNA were detected by NanoDrop 1000 spectrophotometer. The first strand of cDNA was created using RNA as a template, random primers, and reverse transcriptase. The second strand of cDNA was created using the first strand as a template. Reads with joints, lengths less than 50 bp, and average sequence quality below Q20 were filtered out. High-quality sequences were then obtained by *de novo* and reassembling the transcripts. The longest transcripts were chosen as Unigene, and these were subsequently annotated with GO, Kyoto Encyclopedia of Genes and Genomes (Kegg), eggNOG, SwissProt, and Pfam. The filtered sequences were simultaneously aligned to Unigene, and each Unigene’s Reads Count was determined. The samples were then subjected to additional analysis, such as enrichment analysis and expression difference analysis.

### 2.3 Metabolomics analysis

Fresh rhizome tissues (100 mg wet weight) from biennial and triennial *P. cyrtonema* were individually ground with liquid nitrogen into a fine powder. The homogenate was directly resuspended with 500 μL prechilled 80% methanol by vigorous vortexing, without sieving, as the grinding process produced a uniformly fine powder. All samples collected from both groups were of comparable size to ensure consistency. The samples were incubated on ice for 5 minutes and then centrifuged at 15,000 g, 4 °C for 20 minutes. Some of the supernatant was diluted to a final concentration using LC-MS grade water to 53% methanol. The samples were subsequently transferred to a fresh Eppendorf tube and then centrifuged at 15,000 g, 4 °C for 20 minutes. Finally, the supernatant was injected into the LC-MS/MS system analysis.

The hypesil gold column (C18) used in the chromatography process had a column temperature of 40 °C and a flow rate of 0.2 ml/min. Mobile phase B was methanol. While in the positive mode, the mobile phase A was 5 mm ammonium acetate, pH 9.0, and in the negative mode, the mobile phase A was 0.1% formic acid.

The scan range was m/z 100-1500. The ESI source was set as follows: Spray Voltage: 3.2 kV, Sheath gas flow rate: 40 arb, Aux Gas flow rate: 10 arb; Capillary Temp: 320 °C. Polarity: positive, negative. MS/MS secondary scan was data-dependent scans.

Positive ion mode (POS) and negative ion mode (NEG) were used for metabolite coverage and better detection effect.

To compare the data of different orders, the quantitative results were normalized. Finally, the data were identified and the quantitative results were obtained after the raw data had been processed by peak recognition, peak filtering, and peak alignment. Metabolite identification was performed by matching MS/MS spectra against the mzCloud and mzVault databases. Quantification was achieved by integrating peak areas of each metabolite, followed by normalization using internal standards.

The VIP value of the orthogonal partial least squares discriminant analysis (OPLS-DA) and the P value of the t test were combined to screen DEMs between various comparison groups. In the OPLS-DA model, the threshold of difference was VIP 1 and T-test p=0.05.

### 2.4 Analysis of q-PCR

**Figure 1:**
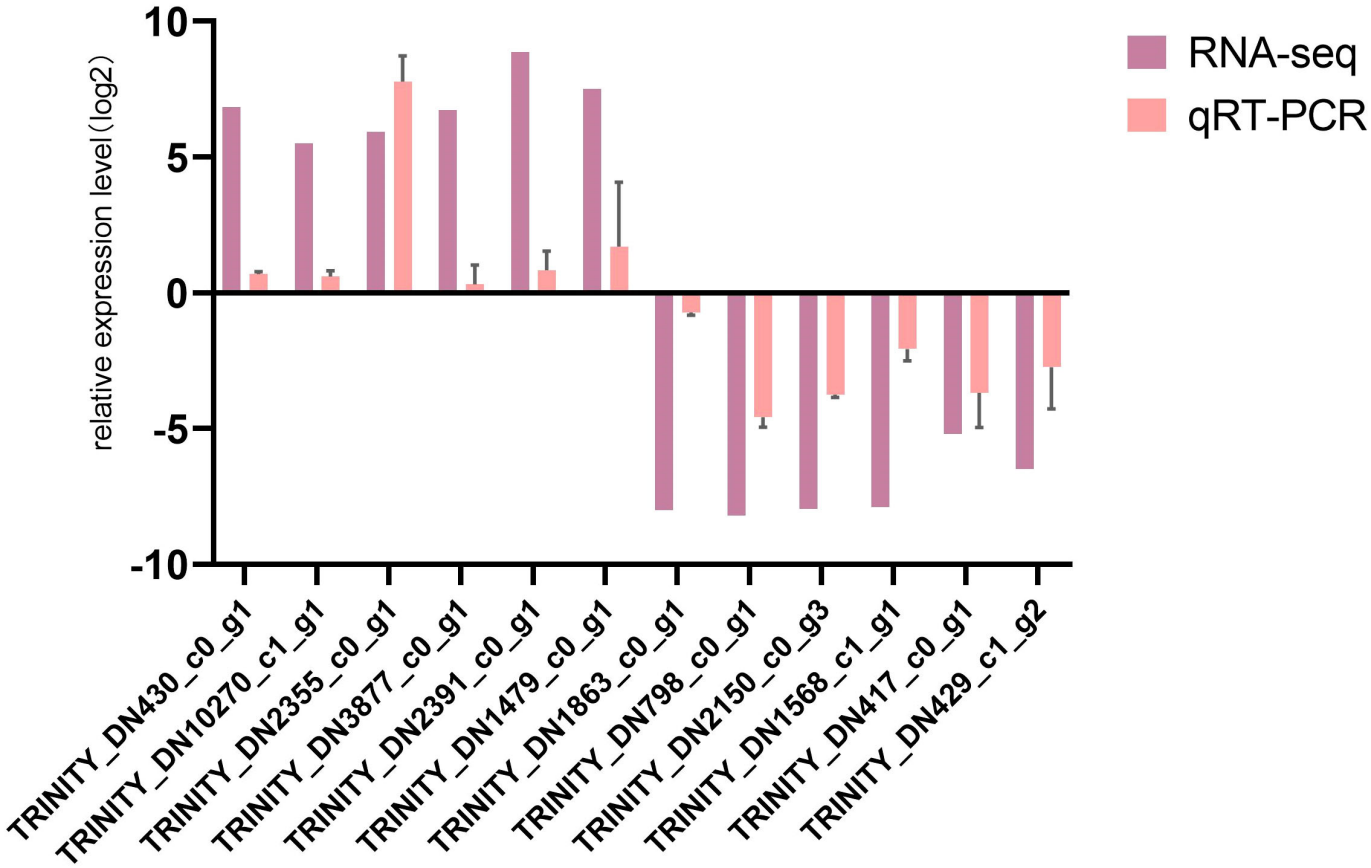
Comparison between q-RT PCR verification results and RNA-seq results.

In order to confirm the accuracy of the transcription data from RNA-seq, 12 DEGs were randomly selected for qRT-PCR validation (primers listed in **Supplementary Table 1**). Each reaction mixture (20 µL) contained 100 ng of cDNA template, 0.5 µM of each primer, and 10 µL of PerfectStart^®^ Green qPCR SuperMix (TransGen). The amplification efficiency of each primer pair was determined by standard curve analysis using a 5-fold serial dilution of cDNA; efficiencies ranged from 90% to 110% (R^2^ > 0.99). The qRT-PCR program was: 94 °C for 30 s, followed by 40 cycles of 94 °C for 5 s, 60 °C for 30 s, and 72 °C for 10 s. All reactions were performed in triplicate. Relative expression levels were calculated using the 2^–ΔΔCt^ method, with β-actin as the internal reference gene. The results are presented as mean ± SD (**Figure 1**). Each qPCR reaction contained 100 ng of cDNA template.

## 3 Results and discussion

### 3.1 Transcriptome sequencing and assembly

Through the transcriptome sequencing of biennial and triennial *P. cyrtonema*, 41,798,602 and 40,249,718 raw reads were obtained with Q30 93.1% and 93.11%, respectively, indicating a greater level of sequencing quality. After filtering the original reads’ quality, 37,857,268 high-quality reads were obtained from the biennial *P. cyrtonema*, and 36894824 clean reads were obtained from triennial *P. cyrtonema* (**Table 1**).

**Table 1:** Summary of the sequence analyses.

Sample	Raw reads	Clean reads	Q20(%)	Q30(%)	Clean reads( %)
B	41798602	37857268	97.32	93.1	90.57
C	40249718	36894824	97.38	93.11	91.66

Sample B: biennial Polygonatum cyrtonema Hua. Sample C: triennial Polygonatum cyrtonema Hua. Clean reads: The read number of high quality sequence. Q20(％): The percentage of bases with more than 99% accuracy in base recognition. Q30(％): The percentage of bases with more than 99.9% accuracy in base recognition.

164,565 transcripts and 69,373 single genes were obtained by using the Trinity program to splice clean readings. The longest transcript is 15,739 bp, and the average length of the transcripts is 1,092.63 bp. The length of N50 was 1556 bp, and the length of N90 was 480 bp, GC content was 43.60% (**Table 2**).

**Table 2:** Sequence overall statistics.

Metric	Transcript	Unigene
Sequence number	164565	69373
Max length (bp)	15739	5739
Mean length (bp)	1092.63	900.85
N50 (bp)	1556	1268
N90 (bp)	480	393

Sequence number: Total number of sequences. Max length (bp): The maximum length of a sequence. Mean length (bp): Average length of sequence.N50 (bp): Arrange all the sequences from long to short, and add the length of the sequence in that order. When the added length reaches 50% of the total length of the sequence, the length of the last sequence. N90 (bp):Arrange all the sequences from long to short, and add the length of the sequence in that order. when the added length reaches 90% of the total length of the sequence, the length of the last sequence.

### 3.2 Unigene function annotation

All 69,373 single genes were compared with NR, GO, KEGG, eggNOG, Swiss-Prot and Pfam databases. 5,453 (7.86%) single genes were annotated in all six databases. The largest number of single genes were annotated to the NR database, with 33,151 (47.79%), and the smallest number of single genes were annotated to the GO database (12,381, 17.85%). 31,870 (45.94%) single genes were annotated to the eggNOG database, 26,226 (37.80%) single genes were annotated to the Swissprot database, 19,417 (27.99%) single genes were annotated to the Pfam database, and 15,693 (22.62%) single genes were annotated to the KEGG database (**Table 3**).

**Table 3:** Summary of functional annotation results.

Database	Number	Percentage
NR	33151	47.79
GO	12381	17.85
eggNOG	31870	45.94
Swissprot	26226	37.80
Pfam	19417	27.99
KEGG	15693	22.62
In all database	5453	7.86

Database: Type of database. Number: The number of Unigene annotated in this database successfully. Percentage: The percentage of successful annotated Unigene to the total Unigene in this database.

According to the species distribution of single genes in NR, 23.93% of single genes matched to oil palm species, 22.11% of single genes matched to *Phoenix dactylifera L.* species, and 7.85% of single genes matched to Musaceae family.

12,381 single genes were annotated into the GO database’s three major categories of biological processes (BP), cellular composition, and molecular function (MF). 6,194 single genes in BP were annotated in the metabolic process, followed by cellular processes. A large number of single genes in CC were enriched in cell and cell parts. Most single genes were annotated for catalytic activity and binding in MF.

### 3.3 Difference analysis

#### 3.3.1 Analysis of DEGs

**Figure 2:**
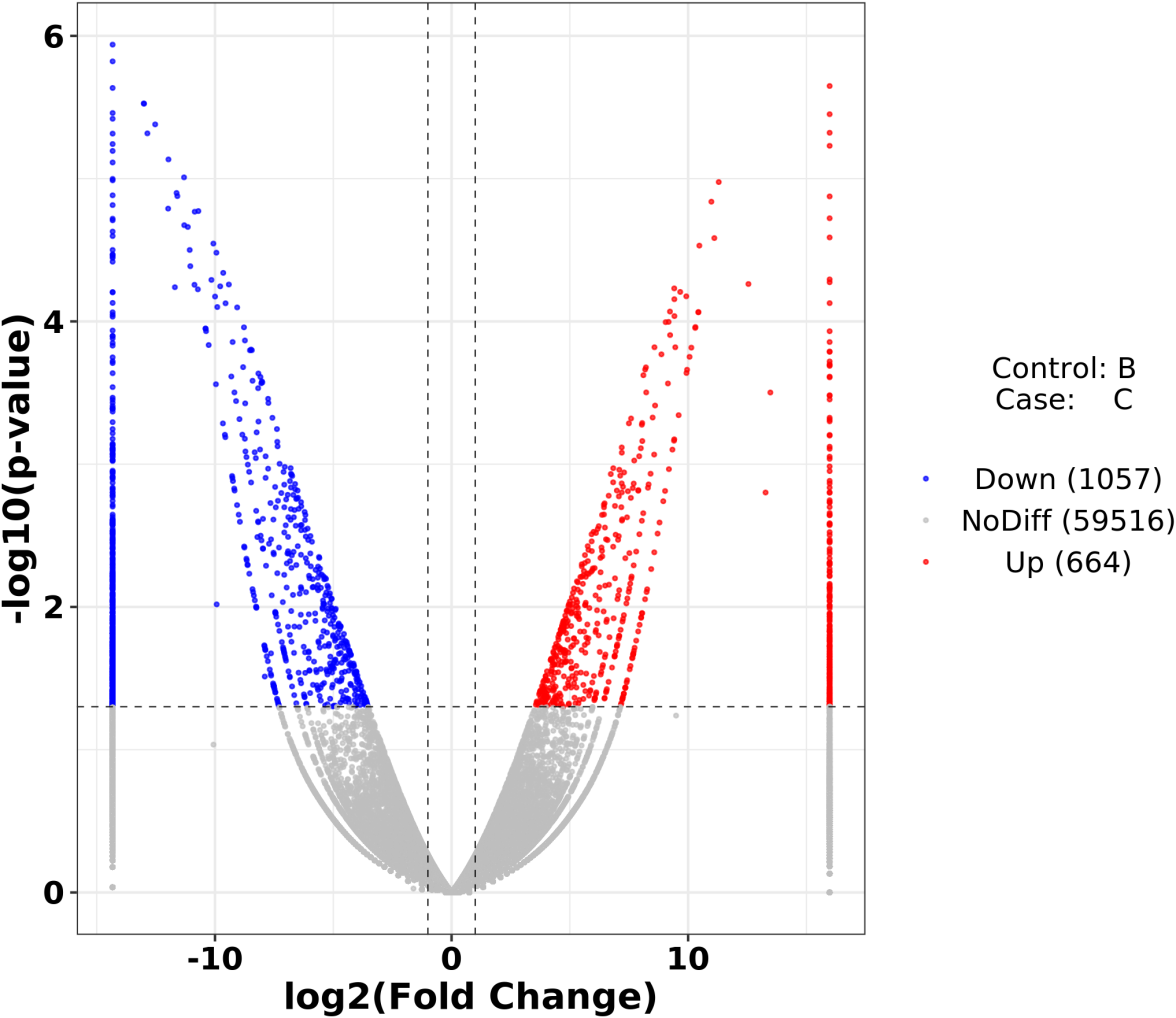
The volcano plot of DEGs. The horizontal coordinate is log2FoldChange and the vertical coordinate is -log10 (p-value). The two vertical dotted lines are 2 times the difference threshold and the horizontal dotted lines are p-value = 0.05. Red dots indicate up-regulated genes, blue dots indicate down-regulated genes, and gray dots indicate non-significantly differentially expressed genes.

Using the transcriptome data of *P. cyrtonema* from biennial and triennial *P. cyrtonema*, the differential gene expression was analyzed by DESeq. The conditions for screening differential gene expression were: |log2FoldChange| > 1, P-value<0.05. 1721 genes were obtained that are differentially expressed, of which 664 genes were up-regulated and 1057 genes were down-regulated (**Figure 2**).

#### 3.3.2 GO enrichment analysis of DEGs

**Figure 3:**
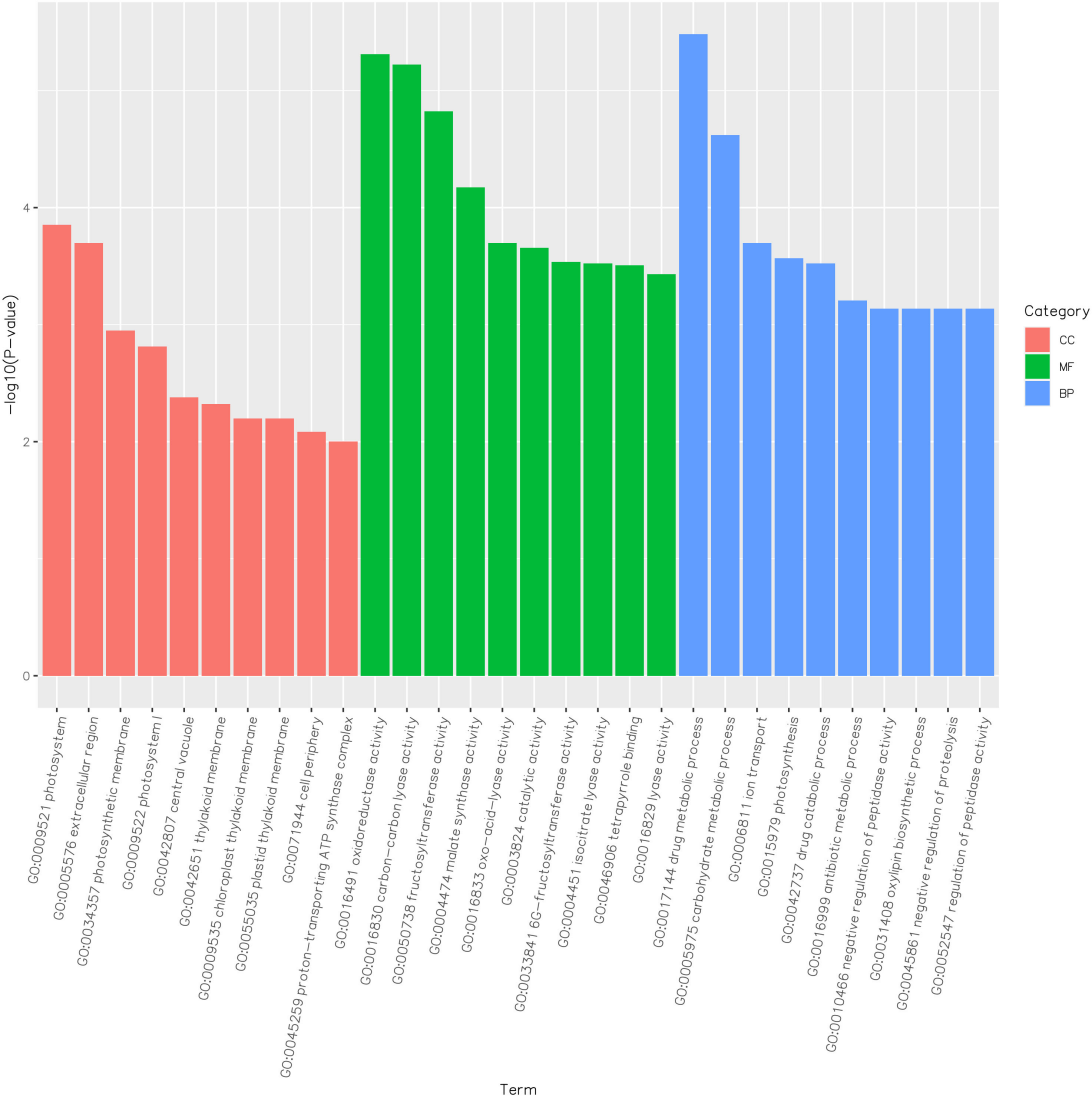
Column chart of GO enrichment analysis. According to molecular function (MF), biological process (BP), and cellular component (CC), GO classification was performed, and the top 10 GO term items with the lowest p-value and the most significant enrichment were selected to present.

**Figure 4:**
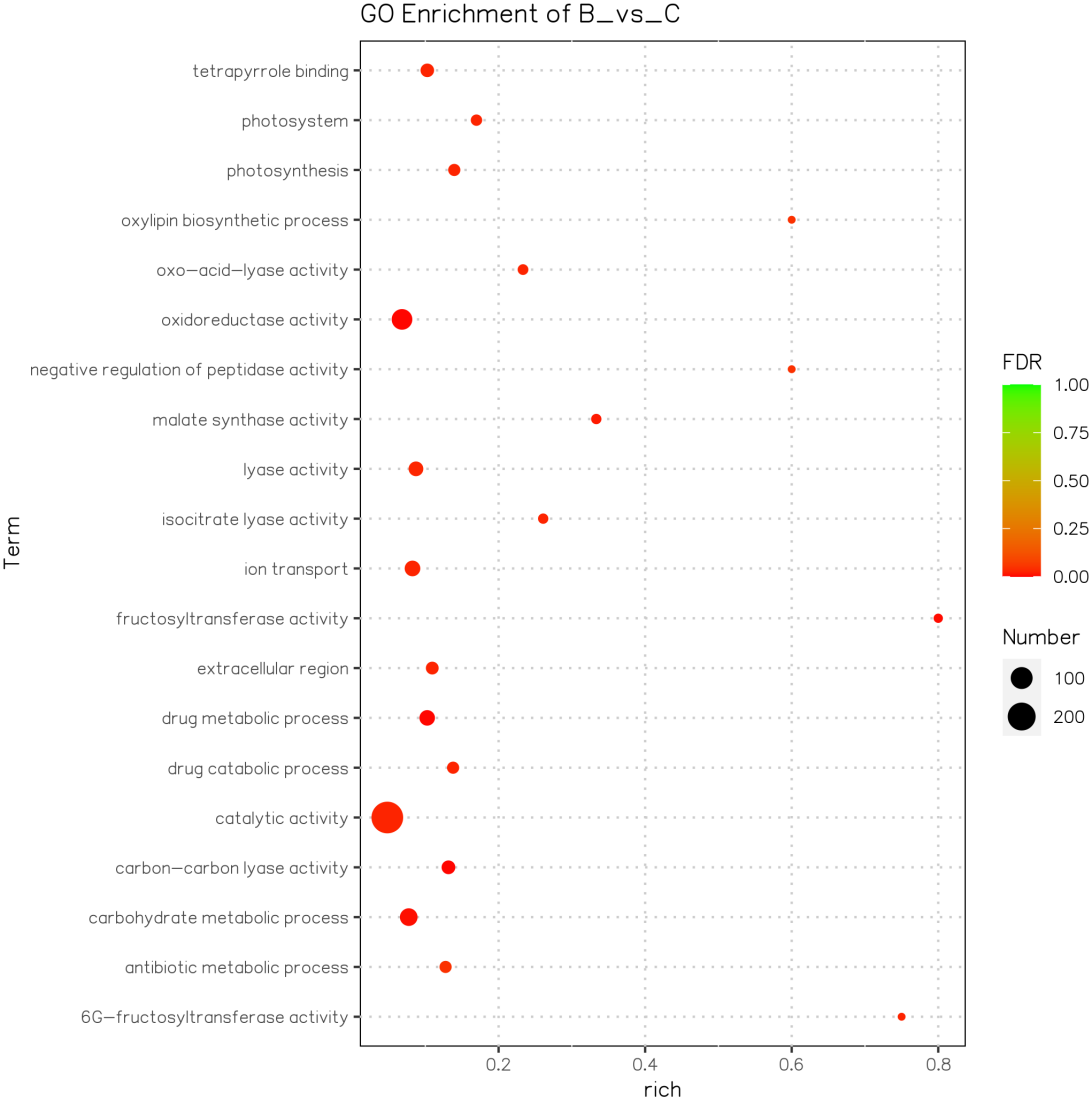
Bubble diagram of GO enrichment analysis. Rich factor refers to the ratio of the number of DEGs enriched in the GO Term to the number of differential genes annotated. The larger the Rich factor, the greater the degree of enrichment. In general, FDR values range from 0 to 1. The more substantial the enrichment, the closer the value is to zero. The figure shows the top 20 GO Term entries with the lowest FDR value and the most significant enrichment.

According to Gene Ontology (GO) enrichment analysis of DEGs, processes enriched in BP included the drug metabolism (GO:0017144), carbohydrate metabolism (GO:0005975), ion transport (GO:0006811), photosynthesis (GO:0015979), drug catabolism (GO:0042737), and antibiotic metabolism (GO:0016999). In CC, many DEGs were enriched in photosystem (GO:0009521), extracellular region (GO:0005576), photosynthetic membrane (GO:0034357), photosystem I (GO:0009522), central vacuole (GO:0042807), thylakoid membrane (GO:0042651), chloroplast thylakoid membrane (GO:0009535), and plastid thylakoid membrane (GO:0055035). In MF, activities that were enriched were found to be oxidoreductase (GO:0016491), carbon-carbon lyase (GO:0016830), fructosyltransferase (GO:0050738), malate synthase (GO:0004474), oxo-acid-lyase (GO:0016833), and catalytic (GO:0003824) (**Figure 3**). According to the FDR, the top 20 GO terms were selected (**Figure 4**). The 10 most significant GO terms on the basis of the GO directed acyclic graph were drug metabolism (GO:0017144), oxidoreductase activity (GO:0016491), carbon-carbon lyase activity (GO:0016830), fructosyltransferase activity (GO:0050738), carbohydrate metabolism (GO:0005975), malate synthaseactivity (GO:0004474), photosystem (GO:0009521), oxo-acid-lyase activity (GO:0016833), ion transport (GO:0006811), and extracellular region (GO:0005576).

#### 3.3.3 KEGG enrichment analysis

**Figure 5:**
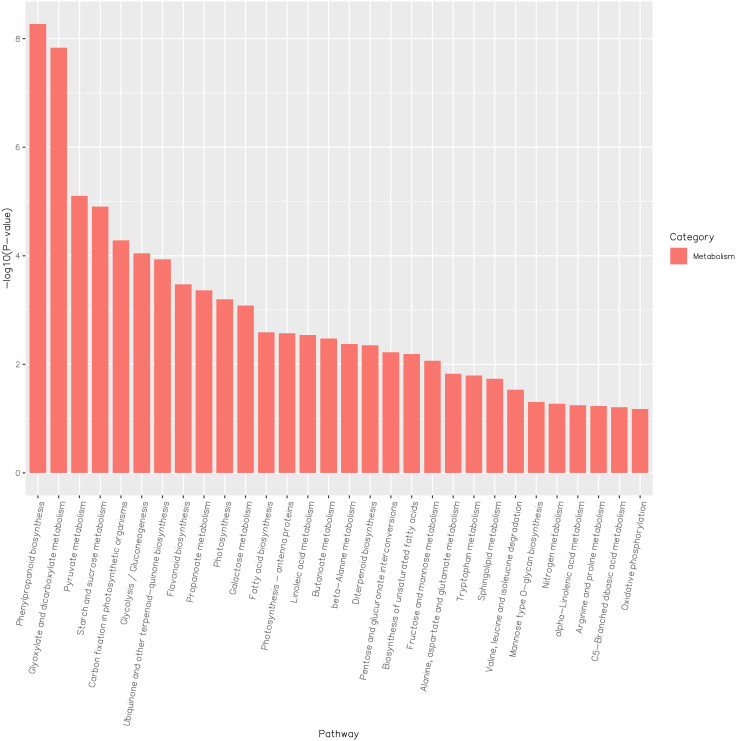
Column chart of KEGG pathway Enrichment result. The top 20 KEGG pathways with the lowest p-value and the most significant enrichment are shown in the Figure.

**Figure 6:**
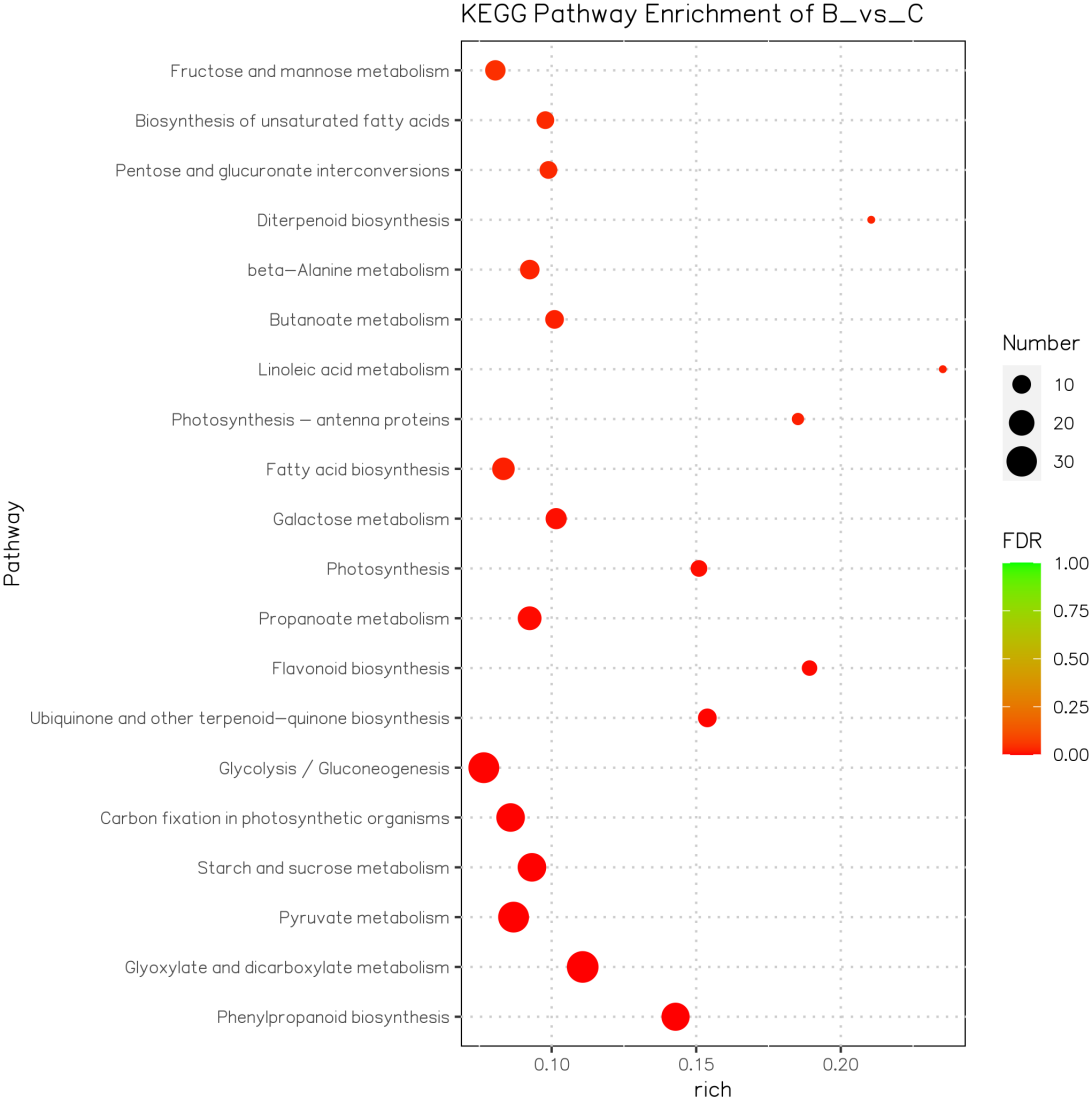
Bubble diagram of KEGG enrichment analysis. The top 20 KEGG pathways with the lowest FDR values are shown.

On the basis of an annotated map of DEGs in KEGG (**Figure 5**), a majority of DEGs were found to be annotated to metabolism pathways. DEGs were most significantly enriched in the phenylpropanoid biosynthesis pathway (ko00940, **Figure 6**), which is central to the synthesis of flavonoids, lignins, and other phenolic compounds—major bioactive components in *P. cyrtonema*. Other enriched pathways included glyoxylate and dicarboxylate metabolism, starch and sucrose metabolism, and flavonoid biosynthesis, indicating that both primary metabolism and secondary metabolite production are influenced by growth years.

#### 3.3.4 KO pathway

In the phenylpropane biosynthesis pathway map, peroxidase was up-regulated in both groups. Based on many pathway maps, some DEGs, such as glycine hydroxymethyl transferase, phosphoenolpyruvate carboxylase, trehalose 6-phosphate phosphatase, endoglucanase, alcohol dehydrogenase 1/7, and chalcone synthase, were up-regulated in triennial *P. cyrtonema*.

#### 3.3.5 Differential transcription factors analysis

**Figure 7:**
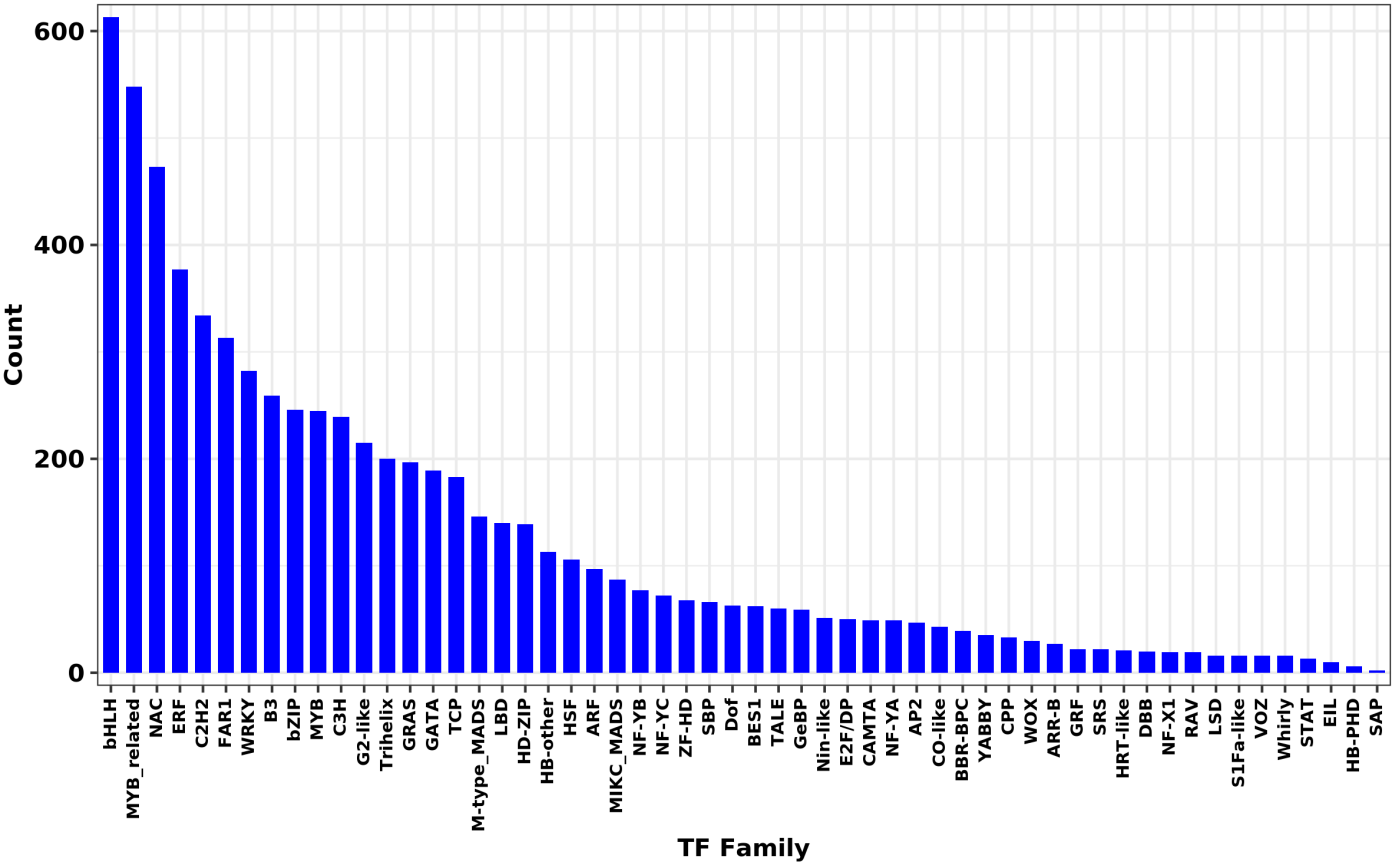
Statistical chart of TFs family. The Abscissa is the different transcription factor family, and the ordinate is the gene number of the transcription factor family.

Among the 56 transcription factor families identified, the most abundant were bHLH, MYB, NAC, ERF, and WRKY (**Figure 7**). These families are known regulators of secondary metabolism, stress responses, and plant development. Their high abundance and differential expression between biennial and triennial plants suggest that they may play key roles in mediating age−dependent accumulation of bioactive compounds.

#### 3.3.6 Validation of DEGs by qRT-PCR

A random selection of 12 DEGs had their expression levels measured using qRT-PCR to confirm the accuracy of the transcription data from RNA-seq (**Figure 1**). The y-axis is relative expression level and the x-axis is biennial and triennial *P. cyrtonema*.

### 3.4 Quality control of metabolic data

**Figure 8:**
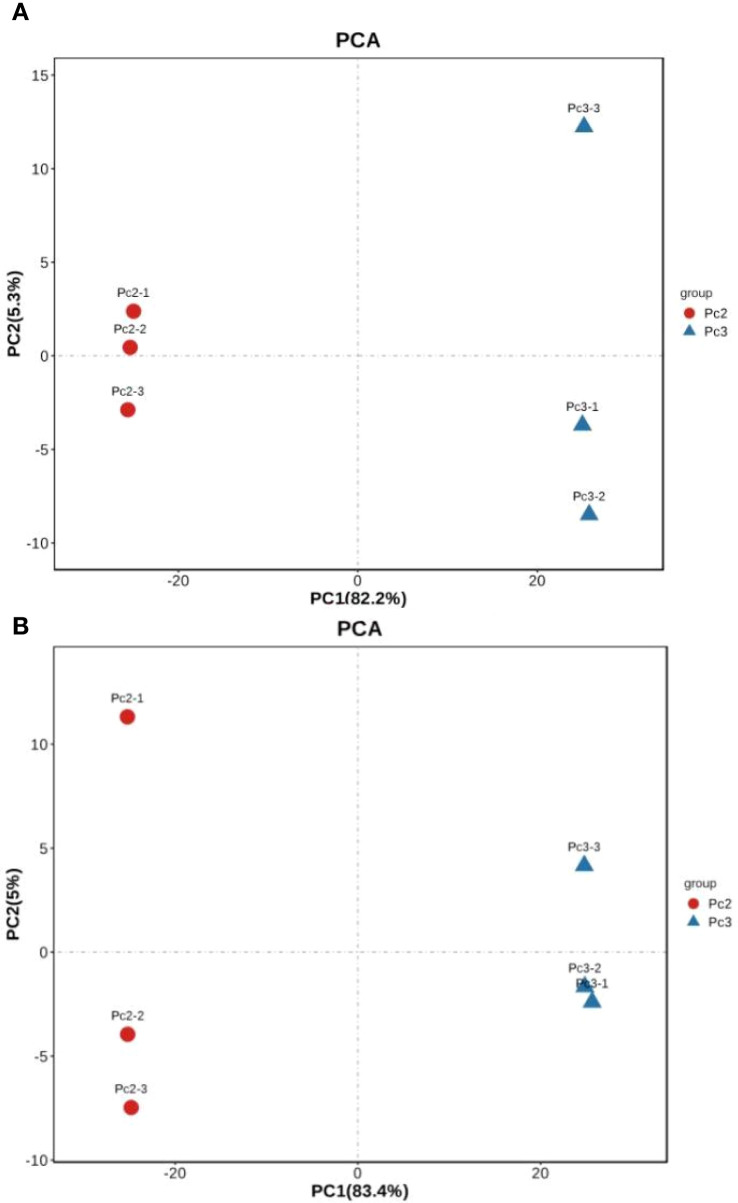
Analysis graphics of PCA. **(A)** PCA analysis of PC2 and PC3 in POS **(B)** PCA analysis of PC2 and PC3 in NEG.

Principal component analysis (PCA) results revealed that *P. cyrtonema* clearly separates on the first and second principle components; samples of PC2 and PC3 were evenly distributed. The detection method has a high level of stability, good data quality, and reliable data (**Figure 8**).

**Figure 9:**
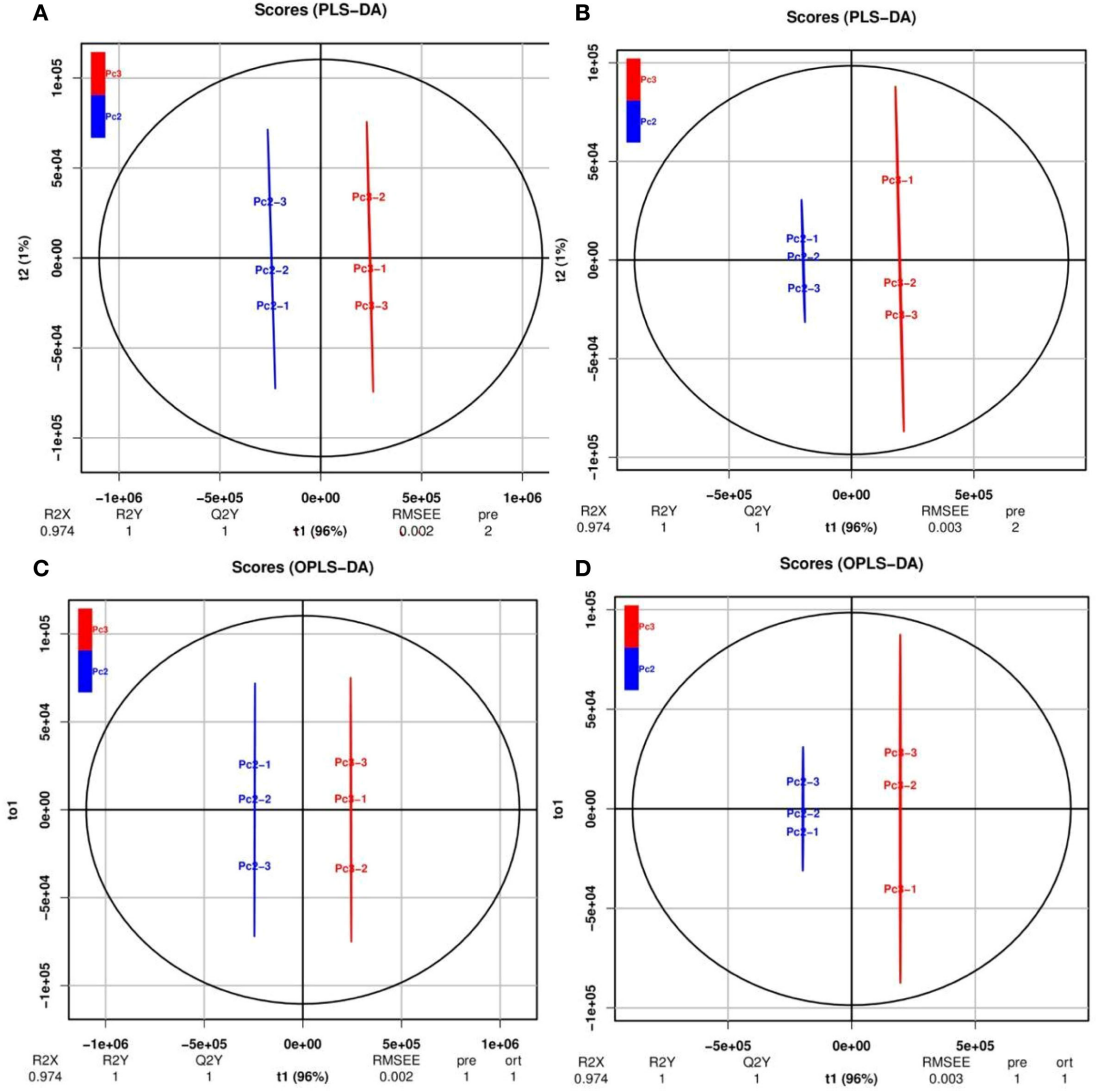
PLS-DA and OPLS-DA analysis. **(A)** PLS-DA analysis in POS **(B)** PLS-DA analysis in NEG **(C)** OPLS-DA analysis in POS **(D)** OPLS-DA analysis in NEG.

Partial least squares discriminant analysis (PLS-DA) and orthogonal partial least squares discriminant analysis (OPLS-DA) plots showed a clear separation between the two groups (**Figure 9**).

### 3.5 Results of metabolite identification

A total of 937 POS ion metabolites were detected, 211 by primary mass spectrometry and 726 by secondary mass spectrometry. 905 NEG ion metabolites were also found, 191 were detected by primary mass spectrometry, and 714 by secondary mass spectrometry.

### 3.6 Analysis of differential metabolites

A total of 150 known DEMs were screened, of which 72 were up-regulated and 78 were down-regulated. These DEMs were analyzed for KEGG pathway enrichment.

**Figure 10:**
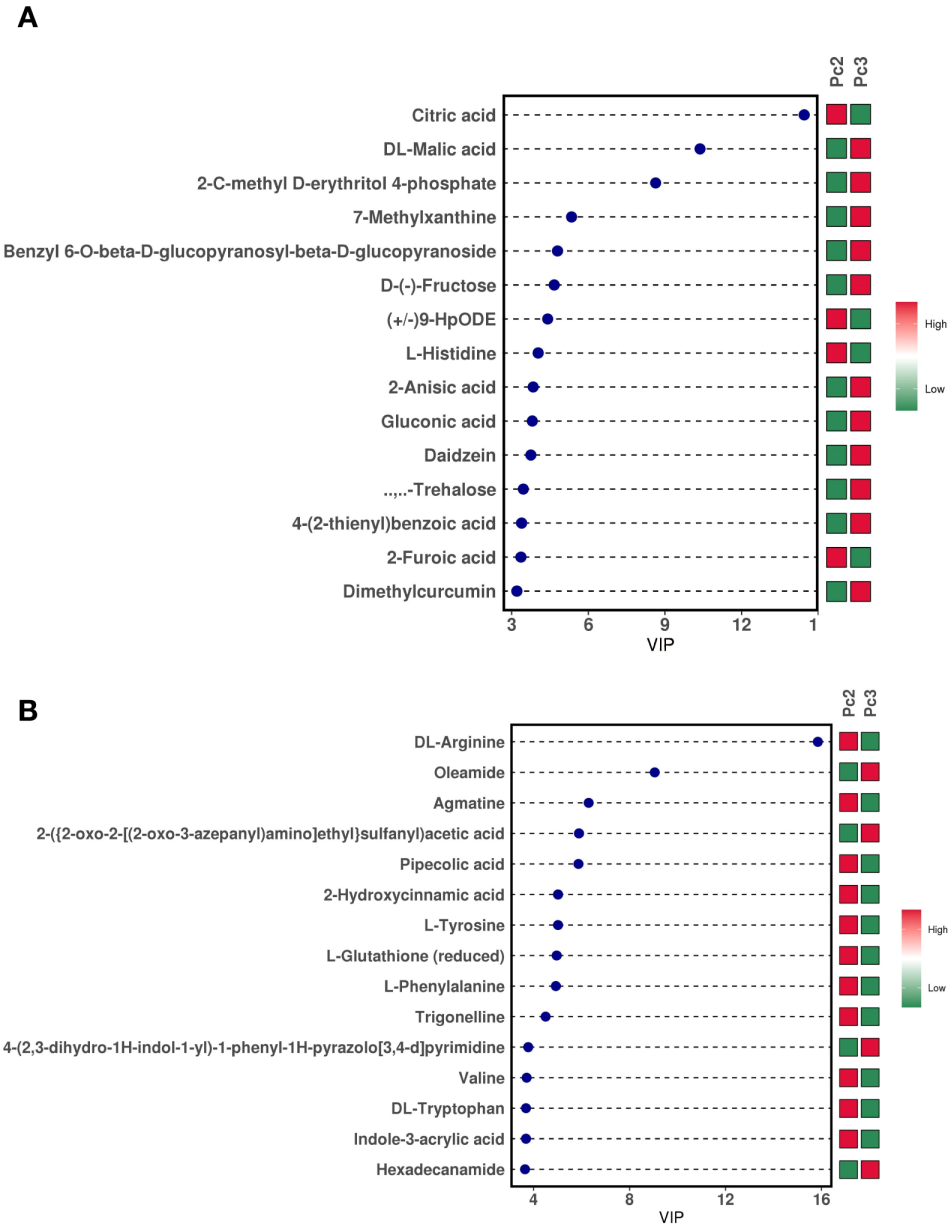
Pictures of VIP-value in OPLS-DA. These two figures show the VIP results corresponding to the differential metabolites (MS2 levels) of the top 15 differential VIP values in each comparison group. **(A)** VIP-value of OPLS-DA in NEG **(B)** VIP-value of OPLS-DA in POS.

In NEG ion mode, the VIP values were greater than 1 for different metabolites in citric acid, DL-malic acid, 2-C-methyl D-erythritol 4-phosphateate, 7-methylxanthine, benzyl 6-o-beta-d-glucopyranosyl-beta-d-glucopyranoside, D- (-) -fructose, (+/-) 9-hpode, L-histidine, 2-anisic acid, daidzein, trehalose, 4- (2-thienyl) benzoic acid, 2-furoic acid and dimethylcurcumin pathways. These metabolites were significantly different between the two groups, and the VIP value of DL-malic acid pathway was the highest. In POS ion mode, the VIP values of different metabolites in DL-arginine, oleamide, agmatine, 2- ((2-oxo-2-((2-oxo-3-azepany) amino) ethyl) sulfanyl) acetic acid, pipecolic acid, 2-hydroxycinnamic acid, L-tyrosine, L-glutathione (reduced), L-phenylalanine, trigonelline, 4-(2,3-dihydro-1H-indol-1-yl) -1-phenyl-1H-pyrazolo (3,4-d) pyrimidine, valine, DL-tryptophan, indole-3-acrylic acid and hexadecanamide were significantly different between the two groups, and the VIP value of DL-arginine pathway was the highest (**Figure 10**).

### 3.7 KEGG enrichment analysis of DEMs

The top 20 pathways with the most enrichment of DEMs were distributed in five KEGG first-level classifications: metabolism, environmental information processing, human diseases, genetic information processing, and drug research and development.

**Figure 11:**
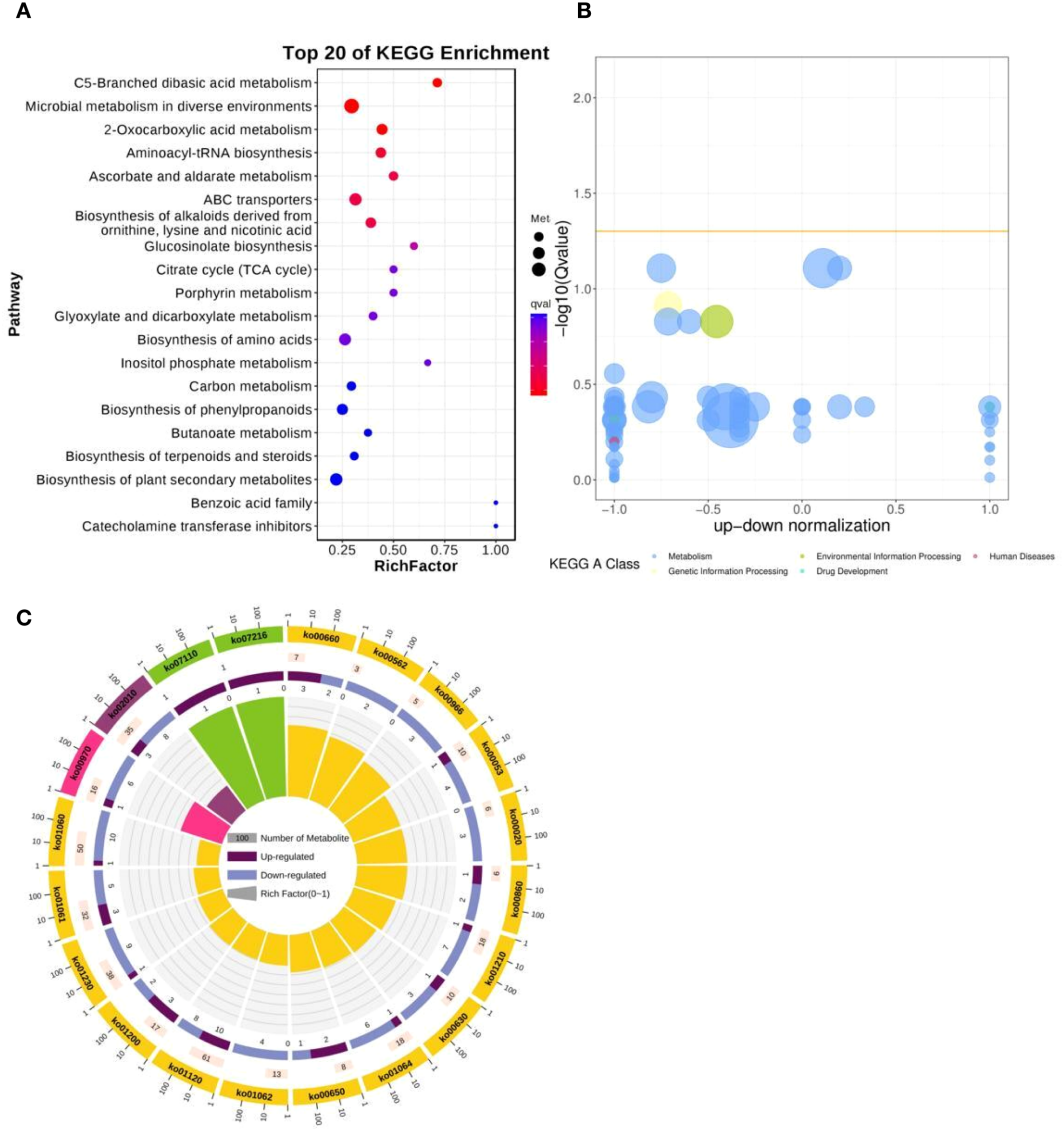
KEGG annotation analysis. **(A)** Enriched bubble map of KEGG. Ordinate is pathway, Abscissa is enrichment factor. The figure shows the top 20 pathway with the lowest Q value **(B)** Bubble map of KEGG differential annotation. The ordinate is-log10 (Qvalue) and the Abscissa is the z-score value. The yellow line represents the threshold of Q-value=0.05. The list on the right is the pathway list of the top 20 Q-value values. Different colors represent different A class. **(C)** Enrichment cycle diagram of KEGG. The first circle: enrichment of the first 20 pathways, outside the circle is the coordinate ruler of the number of differential metabolites. Different colors represent different A class. The second circle: the number and Q-value of the pathway in the background of differential metabolites. The more differential metabolites in the background, the longer the strip shape. The smaller the Q-value, the redder the color. The third circle: up- and down-regulated differential metabolites ratio bar chart. Dark purple represents up-regulated differential metabolites, light purple represents down-regulated differential metabolites, and specific values are shown below. The fourth circle: Rich Factor values of each pathway.

There were 16 metabolic pathways in the top 20 pathways, including C5-branched dibasic acid metabolism (ko00660), inositol phosphate metabolism (ko00562), glucosinolate biosynthesis (ko00966), ascorbate and aldarate metabolism (ko00053), citrate cycle (ko00020), porphyrin metabolism (ko00860), 2-oxocarboxylic acid metabolism (ko01210), glyoxylate and dicarboxylate metabolism (ko00630), biosynthesis of alkaloids derived from ornithine, lysine and nicotinic acid (ko01064), butanoate metabolism (ko00650), biosynthesis of terpenoids and steroids (ko01062), 2−oxocarboxylic acid metabolism (ko1120), carbon metabolism (ko01200), (ko1230), biosynthesis of phenylpropanoids (ko01061), and biosynthesis of plant secondary metabolites (ko01060). There were two drug development pathways: benzoic acid family (ko07110) and catecholamine transferase inhibitors (ko07216), as well as a genetic information processing pathway, aminoacyl-tRNA biosynthesis (ko00970), and an environmental information processing pathway, ABC transporter (ko02010) (**Figure 11**).

### 3.8 Metpa analysis

**Figure 12:**
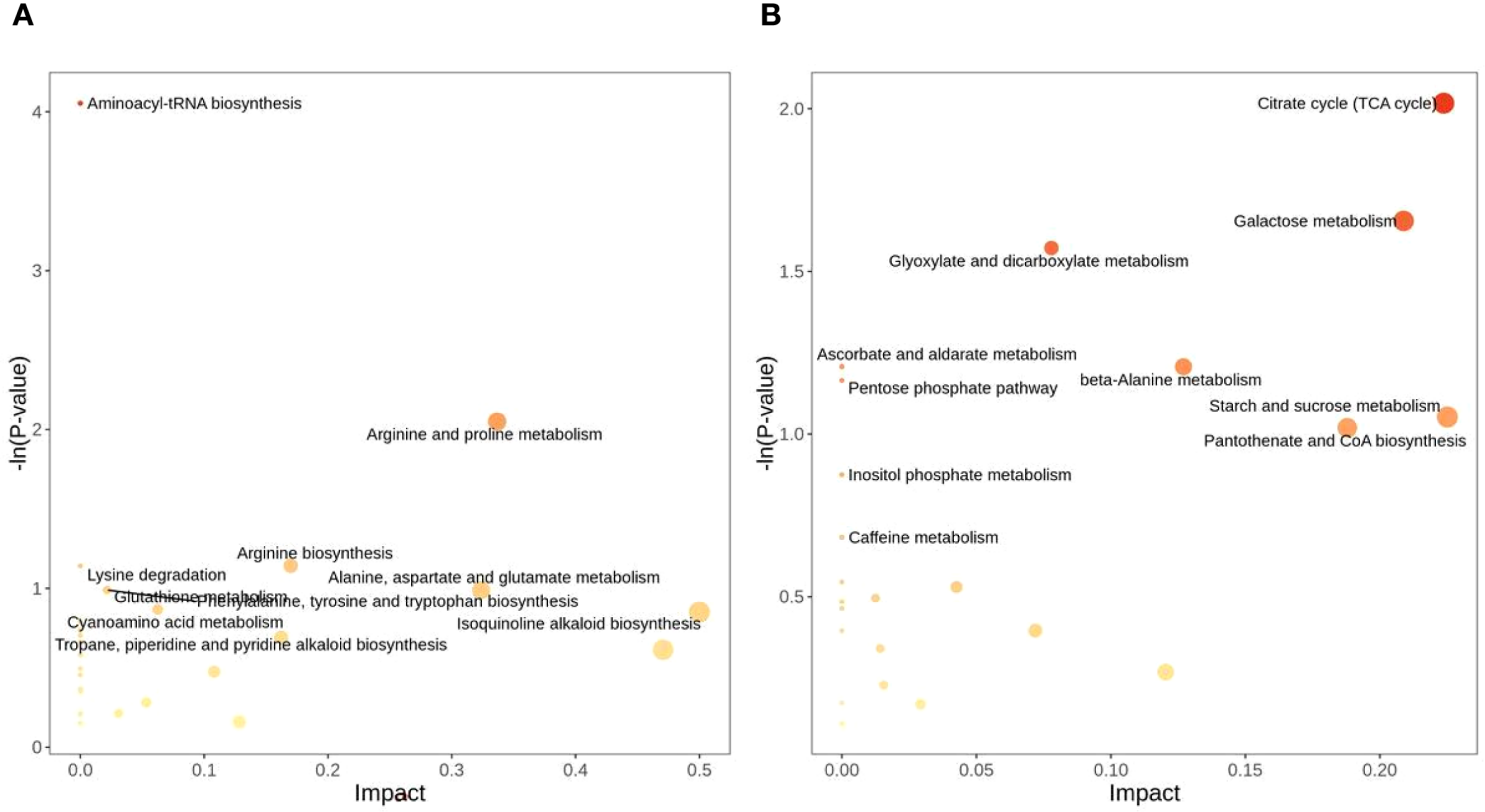
Maps of metpa analysis. **(A)** Map of metpa analysis in POS **(B)** Map of metpa analysis in NEG.

In POS mode, the results of topological analysis demonstrated the significance of many pathways, including aminoacyl-tRNA biosynthesis, arginine and proline metabolism, lysine degradation, arginine biosynthesis, and phenylalanine, tyrosine and tryptophan biosynthesis. The NEG model demonstrated the significance of the tricarboxylic acid cycle, galactose metabolism, glyoxylate and dicarboxylate metabolism, ascorbate and aldehyde metabolism, and beta-alanine metabolism (**Figure 12**).

### 3.9 Co-analysis of transcriptome and metabonomics

The combined multi-omics analysis investigated the biological properties of *P. cyrtonema* at both the genetic and metabolite levels. In addition to contributing towards the foundational knowledge of plant biological systems, the combined analysis of the transcriptome and metabolome can discover significant genes and pathways (Ni et al., 2021; Xu et al., 2023).

Using transcriptomic sequencing and metabolomic analysis, 1721 DEGs and 150 DEMs were identified. Common enrichment pathways biosynthesis of other secondary metabolites, carbohydrate metabolism, glyoxylate and dicarboxylate metabolism, cofactor and vitamin metabolism were present. These pathways are highly interrelated, forming a functional network where the latter three provide the foundational substrates, energy, and cofactors required for the active biosynthesis of secondary metabolites identified as DEMs. In addition to these common pathways other pathways were also related to each other. Thus it can be seen that the growth and development of plants is a complex process, resulting from the common regulation of many genes.

The data of both groups demonstrated that *P. cyrtonema* was enriched in numerous genes under the secondary metabolite biosynthesis pathway. The majority of these genes were enriched in phenylpropanoid biosynthesis and flavonoid biosynthesis.

Phenylpropanoid biosynthesis, a pivotal secondary metabolic pathway in plants, plays a critical role in plant growth, development, and responses to diverse biotic and abiotic stresses by producing key secondary metabolites such as flavonoids, lignin, and anthocyanins (Liu et al., 2021; Lv et al., 2021). Consequently, it represents a major focus in both transcriptomic and metabolomic research. Notably, phenylpropanes are important secondary metabolites in plants due to their role in plant responses to abiotic stresses such as drought, salt and low temperature. For instance, Yu et al, the significant involvement of phenylpropanoid biosynthetic pathway in apple adaptation to freezing stress was revealed through the combined analysis of metabolome and transcriptome. It was found that the phenylpropane biosynthesis pathway and its related pathways are the key pathways for the difference of frost resistance between ‘Ralls’ and ‘Fushi ‘ (Yu et al., 2023). Similarly, Wang et al. discovered through comparative omics analysis that important genes involved in phenylpropanoid biosynthesis, including PAL and 4CL, may increase ROS scavenging activity to support salt or alkali tolerance (Wang et al., 2023). In another study, using combined transcriptome and proteome analyses, Wang et al. identified phenylpropanoid biosynthesis as the primary pathway in Salix mandshurica roots in response to cadmium stress (Yu et al., 2023). Moreover, the functional relevance of this pathway extends to biotic stress: Zhang et al. came to the conclusion that the metabolites produced by the phenylpropanoid biosynthetic route inhibited vibrio dahliae and has potent antibacterial activity (Zhang et al., 2023). Collectively, these findings underscore the versatility and central role of phenylpropanoid biosynthesis in mediating plant adaptation to a wide range of environmental challenges.

Additionally, research suggests that the MYB transcription factor family may affect plant responses to abiotic stressors in tobacco and Arabidopsis through controlling phenylpropanoid biosynthesis (Huang et al., 2016). Additionally, numerous investigations have demonstrated that MYB transcription factor is crucial for the synthesis of phenylpropanoids.

According to the phenylpropanoid biosynthetic pathway map, peroxidase (POD) showed up-regulation in both biennial and triennial *P. cyrtonema*. POD is a redox enzyme widely distributed in plants, animals, and microorganisms and is widely distributed in plants, animals, and microorganisms (de Oliveira et al., 2021). In plants, it is mainly involved in photorespiration, antioxidant defense function, cell wall formation, and metabolism of some organic compounds. POD is not only involved in the physiological and biochemical processes of plants, but also plays an important role in plant stress response and removes reactive oxygen species in cells (Chao et al., 2023). Some studies have shown that POD activity can be affected by stress and other stimuli or changes.

Chalcone is one of the abundant flavonoids found in *P. cyrtonema*. Flavonoids, as a class, are significant secondary metabolites in plant stress resistance due to their roles in antioxidant activity, free radical scavenging, and detoxification of heavy metals (Yao et al., 2019; Xia et al., 2021). Malate dehydrogenase (MDH) is capable of regulating plant growth, tolerance to abiotic stress, and the tricarboxylic acid (TCA) cycle.

Carbohydrate metabolic pathway plays a crucial role in *P. cyrtonema* transcription and metabolic analysis, and it is closely related to the physiological and biochemical processes of plants, such as photosynthesis, synthesis, and decomposition of carbohydrates (Hong et al., 2021; Saddhe et al., 2021). Carbohydrate metabolism involves all the biochemical processes responsible for the formation, breakdown, and transformation of carbohydrates to provide energy to living cells.

The starch and sucrose metabolism pathways, pyruvate metabolism pathways, and glyoxylate and dicarboxylate metabolism pathways are significantly enriched by a number of differential genes under the carbohydrate pathway. They help plants esist adverse environments, strengthen their adaptive ability, and maintain the stability of environmental systems in plants. The initiation of osmotic control and reactive oxygen species (ROS) scavenging, which transport energy in plants, is made possible by the enrichment of glyoxylic and dicarboxylic acid metabolism. An essential process for plant growth and development is the metabolism of galactose, sucrose, and starch. When plants are under stress from the environment, metabolism can provide energy so they can better withstand harm (Zhao et al., 2023). ROS accumulation can be decreased by down-regulating the carbon sequestration pathway (Huang et al., 2021). According to the accumulation of DEMs and the findings of topological analysis, the TCA cycle is a crucial cycle in the polygonatum tricarboxylic acid cycle, which can release and store energy through respiration and supply energy for plants (Zhang et al., 2023).

Amino acids are nitrogen-containing organic compounds and the precursors of many secondary metabolites. They are involved in glycolysis, TCA cycle, and plant stress response. Increased amino acid biosynthesis can provide precursors for stress-related metabolites in plants.

Our analysis identified several transcription factor (TF) families-including bHLH, MYB, NAC, ERF, WRKY, and others-that play important roles in the growth of *P. cyrtonema*. This aligns with the established biological significance of these families. For example, bHLH TFs belong to one of the largest and most extensively dispersed TF families in eukaryotes, and they are known to be crucial for multiple phases of plant development (Liu et al., 2023). *SIPRE3*, a gene that is crucial for plant root development, was found in bHLH by Guo et al. (2023). According to research by Li et al., the bHLH transcription factor *SLBHLH152* regulates Fe homeostasis in plants. Their findings indicated that it is possible for this gene in tomatoes to interact directly with *SLBHLH068* and contribute to the regulation of iron homeostasis (Li et al., 2023). According to Li et al., the CsbHLH gene could react to NaCl, ABA, and low-temperature treatments simultaneously, which enhanced the cucumber’s capacity to withstand abiotic stress (Li et al., 2020). According to Li et al., overexpression of the *AHHLH112* gene in the peanut bHLH transcription factor can increase a plant’s ability to withstand drought and survive drought stress. MYB transcription factors are crucial for the growth and development of plants (Wang et al., 2023). MYB transcription factors are primarily concerned with hormone response, environmental response, and regulation of secondary metabolic pathways. They also actively contribute to sustaining plant development and abiotic stress resistance (Ma et al., 2022). According to certain studies, MYB TFs can regulate some plant metabolic or synthetic pathways in a positive or negative manner (Chen et al., 2023). In strawberries, Zhang et al. found that *FAMYB5* is a positive regulator of anthocyanin and proanthocyanidin production and that MYB transcription factor plays a significant role in the biosynthesis of flavonoids (Zhang et al., 2023). According to Ren et al., overexpressing *VHMYB2* improved *Arabidopsis thaliana*’s resistance to salt and drought stress (Ren et al., 2023). TFs are extensively distributed in plants and play significant roles in tissue formation, plant growth, and stress response.

Implications for medicinal quality and harvest time. The differential expression of phenylpropanoid pathway genes and the corresponding changes in flavonoid-related metabolites suggest that the accumulation of bioactive compounds increases with plant age, peaking at three years under our cultivation conditions. This aligns with traditional knowledge that older *P. cyrtonema* rhizomes are of higher medicinal quality. The identified DEGs and transcription factors (e.g., MYB, bHLH) may serve as molecular markers for optimizing harvest time and improving breeding programs.

## 4 Conclusion

With a rise in societal demand, *P. cyrtonema*, a well-known traditional Chinese medicine with a lengthy history, has been widely cultivated. Growing years are one of several variables that influence the quality and growth of the planting process. This study presents the first integrated transcriptomic and metabolomic profiling of *P. cyrtonema* across different growth years. We identified 1,721 DEGs and 150 DEMs, with the phenylpropanoid biosynthesis pathway as the most significantly enriched and a central regulator of age−dependent active compound accumulation. Key transcription factor families (bHLH, MYB, NAC) that may mediate this process were also uncovered. These findings not only reveal the molecular mechanism linking growth years to medicinal quality but also provide candidate genes and pathways for future molecular breeding and harvest time optimization of *P. cyrtonema*.

## Data Availability

The datasets presented in this study can be found in online repositories. The names of the repository/repositories and accession number(s) can be found below: https://www.ncbi.nlm.nih.gov/genbank/, SRR29139315.
