# Supplementary material for: Transcriptomic and metabolomic profiling of Polygonatum cyrtonema Hua across different growth years
Source: Front Plant Sci. 2026 Jul 1;17:1860542. doi: 10.3389/fpls.2026.1860542 (PMC13368720; doi:10.3389/fpls.2026.1860542)
Supplement: Supplementary file 1 [file SupplementaryFile1.docx]

| Table S1 The genes and primers used for qRT-PCR analysis | | |
| --- | --- | --- |
| Gene ID | Primer pairs | TM value （℃） |
| TRINITY_DN430_c0_g1 | 5'-GGAGGCTCACCGATGTTATTGT-3' | 56.4 |
|  | 5'-TCGGGTAGGGGAAGGGAGAT-3' | 56.1 |
| TRINITY_DN10270_c1_g1 | 5'-TTCAAAGGGGCACATCTAACTG-3' | 53.5 |
|  | 5'-ATATCAAACTCCGCTGCCACTT-3' | 54.5 |
| TRINITY_DN2355_c0_g1 | 5'-CTGCGGAGTTAACTGGTGTGGA-3' | 56.5 |
|  | 5'-CGCGCTCAGATGTCGGTTAGTA-3' | 56.9 |
| TRINITY_DN3877_c0_g1 | 5'-CGACGGGGCGGATGTGAT-3' | 58.7 |
|  | 5'-CCCGGCCGAGGACGAGACG-3' | 64.4 |
| TRINITY_DN2391_c0_g1 | 5'-CCCGGGGTCTGCACTCCTGT-3' | 60.7 |
|  | 5'-AGCTTCTGGCCCGTCGTT-3' | 54.5 |
| TRINITY_DN1479_c0_g1 | 5'-GCCGAGCGAAGGAACTACAAGA-3' | 57.4 |
|  | 5'-CGCCCCGCTGCTAACGAT-3' | 58.3 |
| TRINITY_DN1863_c0_g1 | 5'-CGGCGAAGCAGAGGAGGATTT-3' | 59.3 |
|  | 5'-CGATAGGCCATTGCACCAGTTG-3' | 59.0 |
| TRINITY_DN798_c0_g1 | 5'-TTCGAGTCCGGACAAGAGTTCA-3' | 56.2 |
|  | 5'-GCGCGGGTTTATCAATCCA-3' | 55.7 |
| TRINITY_DN2150_c0_g3 | 5'-TCCCCCGGCAATTCTCCTTTAC-3' | 60.0 |
|  | 5'-TCGTGTTTGGCTTCCTTGATTC-3' | 55.7 |
| TRINITY_DN1568_c1_g1 | 5'-CAAGCAGGCAGATGTGGTGAT-3' | 54.9 |
|  | 5'-GATAGCCCGTCGGATTTTAGC-3' | 54.6 |
| TRINITY_DN417_c0_g1 | 5'-TACCGCCTTGACAGCCATAACC-3' | 57.7 |
|  | 5'-CCTCCCTTCACCTGCTCCATCT-3' | 58.2 |
| TRINITY_DN429_c1_g2 | 5'-CACCCGAAAGGCGAGGAGAATA-3' | 59.1 |
|  | 5'-CCGGACCGTGCCAGAATCAT-3' | 59.2 |
